# Targeted detection and repair of a spinal dural defect associated with successful biochemical resolution of subarachnoid bleeding in classical infratentorial superficial siderosis

**DOI:** 10.1007/s10072-022-06181-x

**Published:** 2022-06-13

**Authors:** Rhannon Lobo, Bilguun Batbayar, Natallia Kharytaniuk, Peter Cowley, Parag Sayal, Simon Farmer, David J. Werring

**Affiliations:** 1 Victor Horsley Department of Neurosurgery, National Hospital for Neurology and Neurosurgery, London, UK; 2 Ear Institute, University College London, London, UK; 3 Department of Neuro-Otology, Royal National ENT and Eastman Dental Hospitals, London, UK; 4 UCLH NIHR Biomedical Research Centre, London, UK; 5 Lysholm Department of Neuroradiology, National Hospital for Neurology and Neurosurgery, London, UK; 6 Department of Neurology, National Hospital for Neurology and Neurosurgery, London, UK; 7 Stroke Research Centre, Department of Brain Repair and Rehabilitation, UCL Queen Square Institute of Neurology, London, UK

**Keywords:** Superficial siderosis, Spinal dural defect, Myelography, Dural repair

## Abstract

**Background and importance:**

Classical infratentorial superficial siderosis (iSS) is characterised by repeated insidious bleeding into the subarachnoid space, leading to haemosiderin deposition within the subpial layers of the brainstem, cerebellum and spinal cord, sometimes with supratentorial involvement. Although nearly always associated with a dural defect (usually from previous trauma or neurosurgery) there is little evidence to support definitive investigation and management strategies. Here, we present a novel investigation strategy to identify a dural defect and subsequent successful surgical repair with biochemical resolution of subarachnoid bleeding.

**Clinical presentation:**

A 55-year-old gentleman presented with a 15-year progressive history of sensorineural deafness, followed by a slowly worsening gait ataxia. He had previously sustained cranio-spinal trauma. On examination there were features of myelopathy and ataxia. MRI demonstrated classical iSS, affecting cerebellum and cerebral cortices, with a cervicothoracic epidural CSF collection. Lumbar puncture (LP) revealed elevated ferritin 413 ng/mL and red cell count of 4160. Reverse CT myelography, a novel technique involving contrast injection into the collection, delineated a dural defect at the T9/T10 level that was not present on conventional myelography. Following surgical repair, repeat LP twelve months later demonstrated biochemical improvement (ferritin 18 ng/mL, red cells < 1). There was no further neurological deterioration in symptoms during eighteen months follow-up.

**Conclusion:**

We show the value of a rational targeted investigation pathway in identifying a surgically reparable dural defect underlying classical iSS. We also provide proof of concept that surgical repair can facilitate biochemical resolution of subarachnoid bleeding and might prevent progression of neurological disability.

**Supplementary Information:**

The online version contains supplementary material available at 10.1007/s10072-022-06181-x.

## Background and importance  

Classical infratentorial superficial siderosis (iSS) is characterised by haemosiderin deposition in the pial layers of the central nervous system, hypothesised causes include, repetitive or sustained low volume bleeding into the subarachnoid space [1, 2]. Whilst iSS can involve the supratentorial cerebral cortices, it is defined by the frequent involvement of typical anatomical regions, including the cerebellum, brainstem, spinal cord and vestibulocochlear nerves. Typically, iSS progresses with irreversible neurological damage manifesting as sensorineural deafness, ataxia and myelopathic signs [3, 4].

The most common cause of iSS, hypothesised to be due to subarachnoid bleeding from a dural defect [4], was referred to by Wilson et al. as type 1, by contrast with type 2 iSS which is associated with a single preceding intra-cranial haemorrhage causing “overspill” of the blood [2]. Cranio-spinal magnetic resonance imaging (MRI) with paramagnetic-sensitive sequences (T2*-weighted gradient echo or susceptibility-weighted imaging) is the initial diagnostic modality of choice. Proposed diagnostic criteria for iSS are the following: “bilateral symmetrical well‐defined curvilinear homogeneous low signal on T2 or blood‐sensitive sequences (…) over the superficial surface of at least 2 of the following regions: brainstem, cerebellum and spinal cord”. Imaging with spinal MRI and then CT myelography (if a dural defect is identified) is recommended. Cerebrospinal fluid (CSF) examination may also support the diagnosis and show ongoing bleeding by demonstrating increased ferritin, red blood cells and xanthochromia. There is a paucity of evidence of efficacy with respect to management; options include iron chelation therapy and surgical repair [2, 5, 6].

Here, we present a case of a classical iSS due to a thoracic dural defect identified by the novel use of reverse dynamic CT myelography; after surgical repair, there was subsequent biochemical improvement of bleeding biomarkers in the CSF with stabilisation of neurological symptoms. This case report builds upon a limited evidence base for thoracic dural repair in classical iSS.

## Clinical presentation

A 55-year-old male builder presented with a 15-year history of progressive bilateral sensorineural hearing loss and a 4-year history of gait ataxia. His background included mild head trauma from a cycling accident at age twelve and a severe blunt-force injury to the thoracic spine 8 years prior to presentation. His injuries at the time did not mandate imaging and he remained asymptomatic for several years. Patient consent for this case report was obtained. He had brisk reflexes throughout with down going plantars and marked gait ataxia. Sensory examination was intact.

**Fig. 1:**
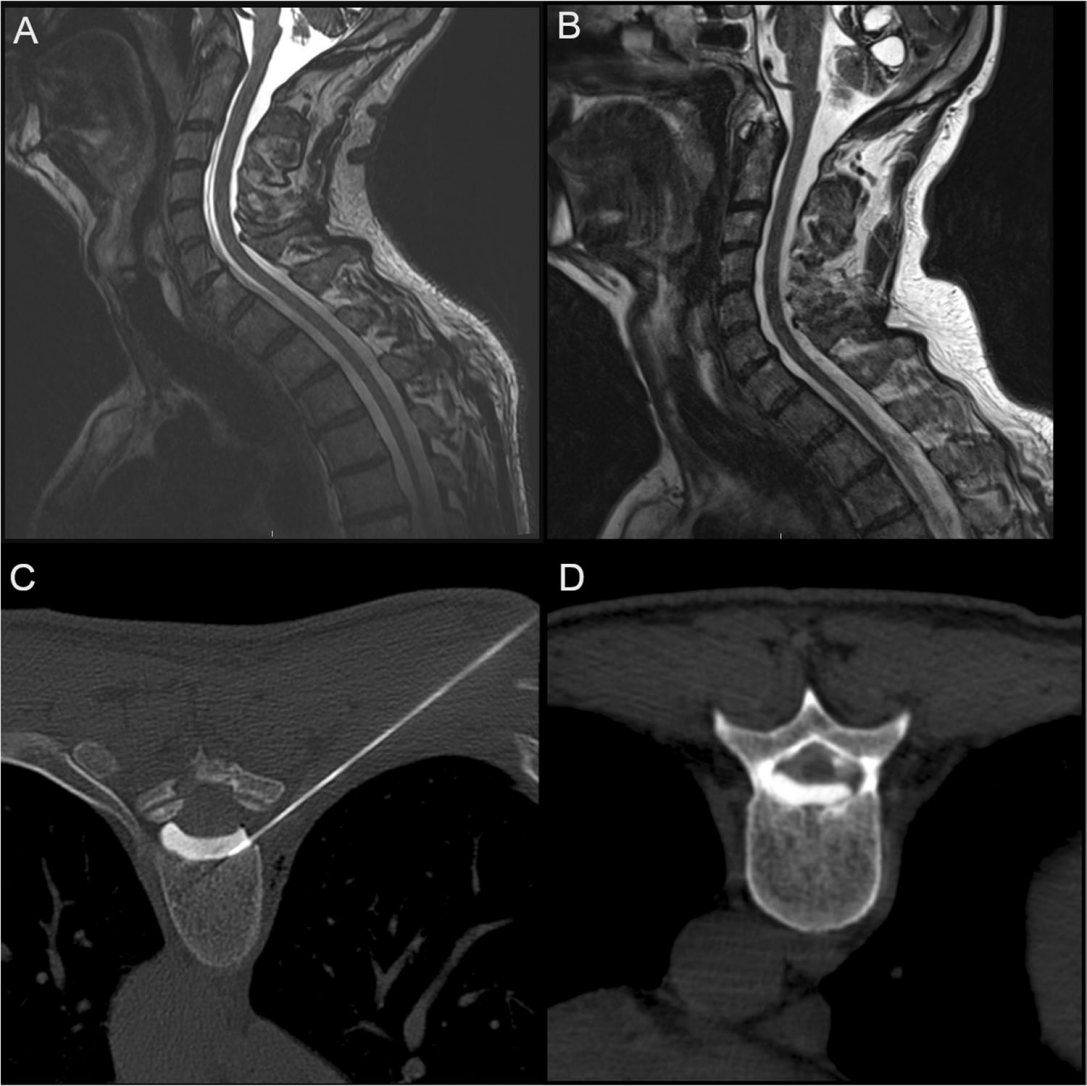
Sagittal MRI spine T2WI SPC revealing a large ventral epidural fluid collection extending from C3 to L3 (**A**) and post-operative resolution (**B**). Reverse dynamic CT myelogram demonstrating contrast injection into the ventral epidural collection (**C**) and subsequent tracking of contrast to the right posterior thoracic cord delineating the site of the dural defect, T9-10 (**D**)

MRI demonstrated extensive siderosis affecting both cerebral and cerebellar hemispheres. The retro-odontoid venous plexus was prominent below C1, suggestive of a CSF leak. No vascular abnormality was identified. Lumbar puncture revealed a CSF ferritin of 413 ng/mL and a red cell count of 4160. MRI spine with contrast demonstrated a ventral cervicothoracic epidural collection (Fig. 1A). Standard and dynamic CT myelography did not identify the exact location of CSF leak. However, reverse CT myelography involving direct contrast injection into the collection demonstrated a ventral dural defect at T9/10 (Fig. 1C, D).

This case was reviewed at the siderosis multidisciplinary team meeting with a decision to repair the dural leak given the marked continued clinical progression and biochemical evidence of ongoing bleeding into the subarachnoid space. He underwent a T8-10 laminectomy with intradural repair of ventral T9-10 dural defect. Intra-operatively, the leak was identified secondary to prolapse of arachnoid through a ventral dural defect between the right T9 and T10 exiting nerve roots. Post-operative imaging performed at 6 months demonstrated resolution of the epidural collection (Fig. 1B) with stable intracranial siderosis. LP was repeated 12 months later, which demonstrated reduction in CSF ferritin to a near-normal value of 18 ng/mL, and absence of any red blood cells. At 18 months, there was no further progression in his neurological symptoms; particularly, his ataxia remained stable.

## Discussion

We report a patient with classical infratentorial superficial siderosis in whom we identified and repaired a thoracic dural defect with post-operative radiological stability, resolution in CSF biochemistry and stabilisation of neurological deterioration. Intra-operatively, we identified and repaired the dural defect at T9–T10 by direct closure, Supplementary Video.

Pertinent points from this case include the need to thoroughly investigate patients with clinical history and imaging concordant with classical type 1 iSS, with rational and targeted radiological tests for a dural defect [2]. In this case, reverse dynamic CT myelography identified the location of the leak. To our knowledge, this is a novel technique that has not previously been described, involving direct contrast injection into the ventral epidural collection.

There is no proven treatment to alter the disease course of iSS. Iron chelation therapy [5, 6] and surgical repair of dural defects [7] are described in small case series and observational studies but there are no controlled trials. Iron chelation with agents such as deferiprone is also associated with clinical stabilisation but have no clear evidence of clinical benefit, and potentially serious side effects such as agranulocytosis and anaemia [8]. Only a few case reports have demonstrated clinical improvement or stabilisation with biochemical resolution of CSF following dural repair in iSS [3, 7, 9, 10].

This case report further supports the use of a rational structured diagnostic algorithm for iSS as previously suggested [2]. Targeted radiological investigation for the site of a dural defect enabled us to treat a patient surgically who may have previously been managed medically without definitive elimination of the presumed bleeding source. Our observation of no further neurological progression at 18 months after surgery is consistent with previous reports that patients with iSS stabilise or improve in neurological function following repair [3, 7, 9, 10]. However, little is known on the long-term benefit and controlled trials are not available. Egawa et al. speculate that dural repair in iSS might be of greater benefit if done as early as possible in the disease course [10].

## Conclusion

In cases where patients present with classical (Type 1) iSS, we recommend reverse spinal CT myelography if conventional imaging fails to identify a dural defect. We suggest that prompt surgical repair might prevent neurological progression. Further research is needed to better establish the risks and benefits of this clinical approach.

## Supplementary Information

Below is the link to the electronic supplementary material.

Supplementary file1 (MP4 86694 KB)

## Declarations

### Disclaimer

The views expressed are those of the authors and not necessarily those of the NHS, the NIHR or the Department of Health.

## References

[1] Fearnley JM, Stevens JM, Rudge P. Review article: superficial siderosis of the central nervous system. Brain. Published online 1995. 10.1093/brain/118.4.1051 10.1093/brain/118.4.1051 7655881

[2] Wilson D, Chatterjee F, Farmer SF, et al. Infratentorial superficial siderosis: classification, diagnostic criteria, and rational investigation pathway. Ann Neurol. Published online 2017. 10.1002/ana.24850 10.1002/ana.24850 28019651

[3] Kumar N, Cohen-Gadol AA, Wright RA, Miller GM, Piepgras DG, Ahlskog JE. Superficial siderosis. Neurology. Published online 2006. 10.1212/01.wnl.0000208510.76323.5b 10.1212/01.wnl.0000208510.76323.5b 16636229

[4] Levy M, Turtzo C, Llinas RH. Superficial siderosis: a case report and review of the literature. Nat Clin Pract Neurol. Published online 2007. 10.1038/ncpneuro0356 10.1038/ncpneuro0356 17205075

[5] Cummins G, Crundwell G, Baguley D, Lennox G. Treatment of superficial siderosis with iron chelation therapy. BMJ Case Rep. Published online 2013. 10.1136/bcr-2013-009916 10.1136/bcr-2013-009916 PMC3736376 23843408

[6] Kessler RA, Li X, Schwartz K, Huang H, Mealy MA, Levy M. Two-year observational study of deferiprone in superficial siderosis. CNS Neurosci Ther. Published online 2018. 10.1111/cns.12792 10.1111/cns.12792 PMC6489821 29285884

[7] Kumar N, Lane JI, Piepgras DG. Superficial siderosis: sealing the defect. Neurology. Published online 2009. 10.1212/01.wnl.0000342457.22536.af 10.1212/01.wnl.0000342457.22536.af 19221303

[8] Flores Martin A, Shanmugarajah P, Hoggard N, Hadjivassiliou M. Treatment response of deferiprone in infratentorial superficial siderosis: a systematic review. The Cerebellum. Published online January 6, 2021. 10.1007/s12311-020-01222-7 10.1007/s12311-020-01222-7 PMC8213658 33409768

[9] Boncoraglio GB, Ballabio E, Erbetta A, Prada F, Savoiardo M, Parati EA. Superficial siderosis due to dural defect with thoracic spinal cord herniation. J Neurol Sci. Published online 2012. 10.1016/j.jns.2011.07.034 10.1016/j.jns.2011.07.034 21868040

[10] Egawa S, Yoshii T, Sakaki K, et al. Dural closure for the treatment of superficial siderosis: report of 2 cases. J Neurosurg Spine. Published online 2013. 10.3171/2013.1.SPINE12649 10.3171/2013.1.SPINE12649 23432322

